# Effects of vibrotactile vestibular substitution on vestibular rehabilitation – preliminary study^☆^^☆☆^

**DOI:** 10.1016/j.bjorl.2015.08.013

**Published:** 2015-09-08

**Authors:** Cibele Brugnera, Roseli Saraiva Moreira Bittar, Mário Edvin Greters, Dietmar Basta

**Affiliations:** a Otoneurology Outpatient Clinic, Hospital das Clínicas, Faculdade de Medicina, Universidade de São Paulo (HCFMUSP), São Paulo, SP, Brazil; b Hospital das Clínicas, Faculdade de Medicina, Universidade de São Paulo (HCFMUSP), São Paulo, SP, Brazil; c Hospital Celso Pierro, Pontifícia Universidade Católica de Campinas (PUC-Campinas), Campinas, SP, Brazil; d Hospital Charité, Berlin University, Berlin, Germany

**Keywords:** Dizziness, Sensory feedback, Vestibular diseases, Postural balance, Rehabilitation, Vertigem, Retroalimentação sensorial, Doenças vestibulares, Equilíbrio postural, Reabilitação

## Abstract

**Introduction:**

Some patients with severe impairment of body balance do not obtain adequate improvement from vestibular rehabilitation (VR).

**Objective:**

To evaluate the effectiveness of Vertiguard™ biofeedback equipment as a sensory substitution (SS) of the vestibular system in patients who did not obtain sufficient improvement from VR.

**Methods:**

This was a randomized prospective clinical study. Thirteen patients without satisfactory response to conventional VR were randomized into a study group (SG), which received the vibrotactile stimulus from Vertiguard™ for ten days, and a control group (CG), which used equipment without the stimulus. For pre- and post-treatment assessment, the Sensory Organization Test (SOT) protocol of the Computerized Dynamic Posturography (CDP) and two scales of balance self-perception, Activities-specific Balance Confidence (ABC) and Dizziness Handicap Inventory (DHI), were used.

**Results:**

After treatment, only the SG showed statistically significant improvement in C5 (*p* = 0.007) and C6 (*p* = 0.01). On the ABC scale, there was a significant difference in the SG (*p* = 0.04). The DHI showed a significant difference in CG and SG with regard to the physical aspect, and only in the SG for the functional aspect (*p* = 0.04).

**Conclusion:**

The present findings show that sensory substitution using the vibrotactile stimulus of the Vertiguard™ system helped with the integration of neural networks involved in maintaining posture, improving the strategies used in the recovery of body balance.

## Resumo

**Introdução:**

Alguns pacientes com déficit severo do equilíbrio corporal submetidos à reabilitação vestibular (RV) podem não apresentar resultados satisfatórios.

**Objetivo:**

Verificar a eficácia do equipamento de biofeedback Vertiguard™ como substituto sensorial do sistema vestibular em pacientes sem bons resultados à RV.

**Método:**

Estudo prospectivo clínico randomizado. Treze pacientes sem resposta satisfatória à RV convencional foram randomizados entre grupo de estudo (GE), que utilizou o estímulo vibratório do Vertiguard™ por dez dias e grupo controle (GC) que usou o equipamento desligado. Para avaliação pré e pós-tratamento foi utilizado o protocolo Teste de Integração Sensorial (TIS) da Posturografia Dinâmica Computadorizada (PDC) e duas escalas de autopercepção do equilíbrio: ABC (*Activities-specific Balance Confidence*) e DHI (*Dizziness Handicap Inventory*).

**Resultados:**

Apenas o GE apresentou melhora estatisticamente significante em C5 (*p* = 0,007) e C6 (*p* = 0,01) da PDC após treinamento. Na escala ABC houve diferença significante no GE (*p* = 0,04). No DHI ocorreu diferença significante no aspecto físico em ambos os grupos e no aspecto funcional (*p* = 0,04) apenas no GE.

**Conclusão:**

O estímulo de substituição sensorial do Vertiguard™ auxiliou a integração das redes neurais e na manutenção da postura, melhorando as estratégias utilizadas na recuperação do equilíbrio corporal.

## Introduction

Postural stability is achieved by the central processing of sensory afferents composed of visual, vestibular, auditory, and proprioceptive information.^1^ The vestibular system, responsible for the integration of this information, determines the appropriate motor response to information incoming from, and outgoing to, environmental demands. The loss of vestibular information sets in motion a structural reorganization of the central nervous system (CNS), that creates new neural networks to replace the lost afferent input.^2^ These changes are responsible for central compensation,^3^ which occurs thanks to neuronal and neurochemical activity caused by sensory conflicts experienced in the absence of vestibular information.^4^ Central compensation may be accelerated by means of vestibular rehabilitation (VR),^5^ which uses physical exercise to restore the main reflexes related to body balance.^6^, ^7^ This concept of neural reorganization in order to address the loss of vestibular function has been termed sensory substitution (SS).^8^

SS can assist in the process of gait and posture stabilization,^9^ by facilitating central compensation of sensory loss, whether partial or complete.^10^ Currently, VR is supported by new man–machine interfaces (MMIs). These interfaces provide stimuli that replace missing natural information, enabling the creation of alternative pathways that act in maintaining balance.^11^ Thus, MMIs are nothing more than alternative stimuli that act on the facilitation of SS. There are descriptions of the additional beneficial effects of neurofeedback in the recovery of body balance with electrotactile stimuli applied to the tongue,^12^, ^13^ auditory biofeedback,^14^, ^15^ and audiovisual biofeedback.^16^ However, Basta and Ernst^17^ believe in the effectiveness of using vibrotactile biofeedback; with this, the subject is not deprived of the natural perception of sound and visual stimuli from the environment. Studies have shown the effectiveness of vibrotactile biofeedback equipment applied on the lateral aspect of the trunk, with increased postural stability^18^ and improved alignment of the center of mass.^19^

In a controlled double-blinded pilot study, 36 patients divided into five groups with vestibular disorders of different etiologies showed significant reduction in body oscillation after training with the Vertiguard™ device.^20^ Another study with 105 patients suffering from balance disturbances showed a reduction in their symptoms only in the study group, with decrease in anteroposterior oscillation, increase in balance index value, and in conditions 5 and 6 of the Sensory Organization Test (SOT) protocol of Computerized Dynamic Posturography (CDP).^21^

The aim of this study was to evaluate the effectiveness of vibrotactile neurofeedback (Vertiguard™) as a sensory substitute in patients who did not obtain a good response to conventional VR.

## Methods

This study was approved under No. 0896/09 by the Institutional Ethics Committee. All participants signed an informed consent prior to their inclusion in the study. They are part of the initial sample, 15 subjects with vestibular disorders who did not achieve satisfactory results after following a conventional protocol of vestibular rehabilitation. Neurological and/or orthopedic limitations that prevented the realization of CDP or training with the Vertiguard™ were considered as exclusion criteria. The subjects were randomly assigned to a study group (SG) (who received the vibration during training) and a control group (CG) (who trained with the power off). The same therapist performed all training; orders and directions were exactly the same for both groups.

Patients included in the study followed a protocol of anamnesis, otorhinolaryngological examination, static (Romberg) and dynamic (Fukuda) stability tests, coordination tests, audiometry, acoustic impedance, electro-oculography with caloric stimulation using water (at 44 °C and 30 °C; and at 18 °C when there were no responses to the foregoing temperatures), and the SOT protocol of the CDP (Equitest NeuroCom™).^22^

Next, the subjects answered the Brazilian version of two assessment questionnaires, the Dizziness Handicap Inventory (DHI)^23^ and the Activities-specific Balance Confidence (ABC) Scale.^24^, ^25^

The DHI assesses and quantifies the impact of dizziness on quality of life of the patient. It consists of three separate assessments distributed among 25 questions; seven assess physical aspects (worsening of dizziness as a result of movements or actions); nine consider the functional aspects (limitation of activities of daily living by dizziness); the final nine questions assess the emotional aspects (loss of social life or feelings of insecurity, fear, depression caused by dizziness). The patient must respond to all 25 questions, using ‘yes’, which corresponds to 4 points; ‘sometimes’, which corresponds to 2 points, or ‘no’, which corresponds to zero points. Therefore, the higher the score, the worse the quality of life.

The ABC scale quantifies the self-perception level of body balance disorders and helps in selecting appropriate interventions. The patient quantifies (in percentage, from zero to 100%) his/her self-confidence in performing 16 tasks of daily living. Therefore, the higher the percentage, the higher his/her self-confidence.

**Figure 1:**
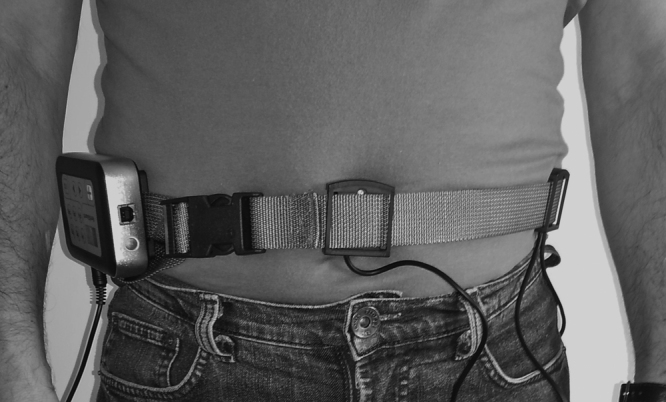
Vertiguard™ coupled to the patient's waist.

**Figure 2:**
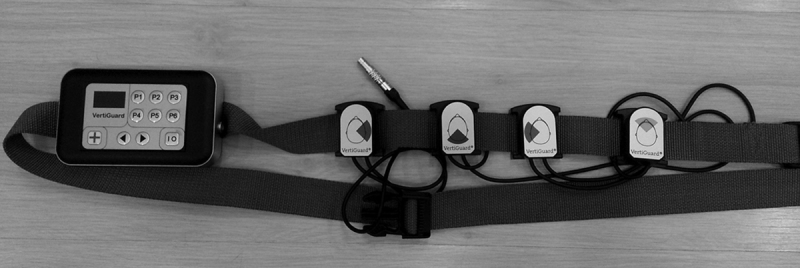
Vertiguard™: main unit and vibrotactile stimulators.

After evaluation, patients were trained with Vertiguard™. The device has body balance assessment and training functions. It consists of an adjustable belt placed around the patient's waist (Fig. 1) containing a main unit fitted with two gyroscopes, which detect the direction of the body oscillation (anterior/posterior, R/L side), and four vibrating stimulators arranged at angles of 90° between them (Fig. 2). These vibrating units respond to the command of the main unit to produce vibration, signaling the direction of body displacement.

The assessment aims to quantify, according to an established normal standard, the body movements of the subject when performing various static and dynamic tasks, namely: patient standing with eyes open and closed; supported on one foot with eyes open; supported on one leg with eyes closed (only for patients younger than 60 years); marching eight steps, touching the heel with the big toe (tandem gait); standing with open and closed eyes on a foam surface; supported on one foot with eyes open; marching eight steps, touching the heel to the big toe (tandem gait) on a foam surface; walking 3 m; walking 3 m performing circular movements of the head; walking 3 m moving head up and down; walking 3 m with closed eyes; skipping a sequence of six obstacles; and sitting down and standing up from a chair (for subjects over 60 years). When used in the training mode, the main unit selects and stores the six worst responses obtained in the previous evaluation, to be trained over a period of ten days, according to the protocol established when the equipment was developed at the University of Berlin. While performing the selected tasks, if any inappropriate body deviation occurs, the stimulators emit vibratory signals indicating to the subject the direction of his/her movement. Thus, the equipment helps in the perception of inappropriate movements, replacing the lost vestibular information.

The clinical effect of the treatment was determined by a comparison between the results of evaluations performed before and after the treatment with the conditions 5 and 6 of the SOT protocol of the CDP – conditions typically considered as vestibular. The responses of the two scales of assessment were also compared, in order to determine the self-perception of the patient regarding his/her improvement.

The difference between the posturographic values obtained before and after treatment, considering that they follow a normal distribution, was determined by paired Student's *t*-test and considered significant when *p* < 0.05. For DHI and ABC scales, the Kruskal–Wallis test was used – a particularly suitable test, because of the small samples.

## Results

Fifteen subjects agreed to participate in the study (ten men and five women, age 71.3 ± 10.8 years). During the training process, two patients could not complete the protocol due to health issues, reducing the final sample to 13 individuals, seven in the SG and six in the CG. Table 1 shows the age, gender, vestibular function, and etiology of the subjects in the sample.

**Table 1:** Presentation of CG and SG according to age, gender, vestibular function, and etiology of body imbalance.

Age	Gender	Labyrinthine function	Etiology
*CG*			
55	M	Bilateral hyporeflexia	Seizure
73	M	Bilateral hyporeflexia	VBI
79	F	Bilateral hyporeflexia	ISE
87	F	Normoreflexia	Metabolic/ISE
82	F	Unilateral areflexia	Metabolic
55	M	Unilateral areflexia	Sudden deafness

*SG*			
84	F	Bilateral hyporeflexia	Idiopathic
62	M	Bilateral hyporeflexia	Right labyrinth fracture
64	M	Bilateral hyporeflexia	Trauma
77	M	Normoreflexia	Metabolic
79	M	Bilateral hyporeflexia	ISE
64	M	Bilateral areflexia	Idiopathic
67	M	Bilateral hyporeflexia	Hepatitis C

CG, control group; SG, study group; VBI, vertebral-basilar insufficiency; ISE, imbalance syndrome of the elderly; TBI, traumatic brain injury. Areflexia was considered as the absence of post-caloric responses; hyporeflexia as angular velocities <4°/s.

The values obtained in posturography conditions C5 and C6 and on the ABC and DHI scales in the beginning of the experiment showed no statistical difference between the CG and SG, characterizing these as homogenous groups.

Between pre- and post-treatment means, a significant difference for C5 (*p* = 0.007) and C6 (*p* = 0.012) was found only in the SG. The pre- and post-treatment of the C5 and C6 are shown in Table 2.

**Table 2:** Mean values for C5 and C6 before and after training in the CG and SG.

Group	C5 pre-	C5 post-	*p*	C6 pre-	C6 post-	*p*
CG	34.83	48.88	0.098	43.33	54.05	0.165
SG	26.85	53.23	0.007^a^	15.85	41.19	0.012^a^

CG, control group; SG, study group.

a Statistical significance.

Among the responses to DHI questionnaire before and after treatment, a significant difference in the physical aspect in the CG (*p* = 0.0400) and the SG (*p* = 0.0423) was observed; and in functional aspect, only in the SG (*p* = 0.0427). The results found are shown in Table 3.

**Table 3:** Numerical values of means obtained on the Dizziness Handicap Inventory (DHI) scale before and after training by the CG and SG.

DHI	Pre-physical	Post-physical	*p*	Pre-functional	Post-functional	*p*	Pre-emotional	Post-emotional	*p*
CG	17.66	10.33	0.04^a^	19.33	14.33	0.09	15.66	10.66	0.21
SG	13.71	5.42	0.04^a^	18.85	7.71	0.04^a^	15.71	7.71	0.14

CG, control group; SG, study group.

a Statistical significance.

The results for ABC scale demonstrate a significant difference between the beginning and end of the treatment in the SG (*p* = 0.04) but not in the CG (*p* = 0.12). The numerical values are shown in Table 4.

**Table 4:** Percent values of means obtained on the Activities-specific Balance Confidence (ABC) scale before and after training by CG and SG.

ABC	Pre-	Post-	*p*
Control group	58.84	69.68	0.12
Study group	68.66	88.03	0.04^a^

CG, control group; SG, study group.

a Statistical significance.

## Discussion

The results of this study suggest that Vertiguard™ vibrotactile stimulation device was able to improve the body balance of patients who did not achieve good response to training by vestibular rehabilitation. While VR is recognized as an effective method for training subjects who present with seriously impaired postural stability, its results are still limited in these cases, and other methods that seek sensory substitution have been studied.

The present sample was composed of elderly individuals (mean age 71.3 years) who clearly had severe postural impairment, which represents the reason they did not achieve improvement with conventional VR. It can be observed that, even with larger numbers of subjects with bilaterally compromised labyrinthine function, only SG subjects, who received the vibrotactile stimulation, showed a statistically significant improvement in conditions C5 and C6. This result suggests that the additional vibrotactile stimulus was able to stabilize the posture under those challenging conditions encountered during training.

Conditions C5 and C6 of the SOT protocol of the CDP are called vestibular conditions because, on unstable ground with absent (C5) or conflicting (C6) vision, postural maintenance depends exclusively on vestibular information. The commitment to this information causes exaggerated postural oscillation, or falls. The improvement achieved by the group treated with vibration (SG) suggests that the CNS used the additional stimulus to integrate extra information and improve its postural recovery strategies.

Addressing assessment scales, improvement of the functional aspect of DHI was noted in the group treated with vibration during training. This result implies that only the subjects in the treated group showed a reduction of interference from their dizziness in their daily tasks after this therapeutic approach, demonstrating the effectiveness of the training associated with sensory substitution. Patients felt safer in performing their activities despite their physical limitations. With regard to the physical aspect of DHI, it is useful to note that both the SG and the CG showed improvement of their indices after the intervention. These results can be explained by the intensive physical exercise performed during the period by subjects previously suffering restriction due to their limitation of movement. In contrast, neither group achieved changes in the emotional aspect between pre- and post-training phases. Therefore, there was no difference in the emotional impact caused by dizziness in their life after the intervention.

The level of self-confidence, measured by the ABC questionnaire, improved significantly only in the group receiving the vibrotactile stimulus. Again, these results suggest the beneficial impact of vibrotactile stimulation associated with vestibular rehabilitation in the recovery of cases with severely impaired postural control.

## Conclusion

This was a preliminary study, conducted with a small number of patients, but it already has shown unquestionably that vibrotactile biofeedback, as a sensory substitution to the vestibular system, is a useful tool in patients with limitations in their postural recovery with conventional vestibular rehabilitation protocols.

## Funding

This study was funded by a 10.13039/501100002322CAPES research grant from July 2011 to June 2013.

## Conflicts of interest

The authors declare no conflicts of interest.
